# argNorm: normalization of antibiotic resistance gene annotations to the Antibiotic Resistance Ontology (ARO)

**DOI:** 10.1093/bioinformatics/btaf173

**Published:** 2025-04-16

**Authors:** Svetlana Ugarcina Perovic, Vedanth Ramji, Hui Chong, Yiqian Duan, Finlay Maguire, Luis Pedro Coelho

**Affiliations:** Institute of Science and Technology for Brain-Inspired Intelligence, Fudan University, Shanghai, 200433, China; Department of Cellular, Computational and Integrative Biology – CIBIO, University of Trento, Trento, 38123 Povo, Italy; APL Global School, Chennai, 600097, Tamil Nadu, India; Centre for Microbiome Research, School of Biomedical Sciences, Queensland University of Technology (QUT), Translational Research Institute, 37 Kent St., Brisbane, Queensland, 4102 Woollongabba, Australia; Institute of Science and Technology for Brain-Inspired Intelligence, Fudan University, Shanghai, 200433, China; Institute of Science and Technology for Brain-Inspired Intelligence, Fudan University, Shanghai, 200433, China; Faculty of Computer Science, Dalhousie University, Halifax, Nova Scotia, B3H 4R2, Canada; Department of Community Health & Epidemiology, Faculty of Medicine, Dalhousie University, Halifax, Nova Scotia, B3H 4R2, Canada; Institute of Science and Technology for Brain-Inspired Intelligence, Fudan University, Shanghai, 200433, China; Centre for Microbiome Research, School of Biomedical Sciences, Queensland University of Technology (QUT), Translational Research Institute, 37 Kent St., Brisbane, Queensland, 4102 Woollongabba, Australia

## Abstract

**Summary:**

Currently available and frequently used tools for annotating antibiotic resistance genes (ARGs) in genomes and metagenomes provide results using inconsistent nomenclature. This makes the comparison of different ARG annotation outputs challenging. The comparability of ARG annotation outputs can be improved by mapping gene names and their categories to a common controlled vocabulary such as the Antibiotic Resistance Ontology (ARO). We developed argNorm, a command line tool and Python library, to normalize all detected genes across six ARG annotation tools (eight databases) to the ARO. argNorm also adds information to the outputs using the same ARG categorization so that they are comparable across tools.

**Availability and implementation:**

argNorm is available as an open-source tool at: https://github.com/BigDataBiology/argNorm. It can also be downloaded as a PyPI package and is available on Bioconda and as an nf-core module.

## 1 Introduction

Antibiotic resistance is a major public health concern (Antibiotic Resistance Threats in the United States 2019, Singh *et al.* 2021, Murray *et al.* 2022) that is partially mediated by the presence of antibiotic resistance genes (ARGs) in microbial genomes (Blair *et al.* 2015, Zhang *et al.* 2022). Given this, several methods and tools have been developed for annotating ARGs in genomes and metagenomes (each with their strengths and weaknesses) (Bortolaia *et al.*, Gupta *et al.* 2014, Arango-Argoty *et al.* 2018, Rowe and Winn 2018, Feldgarden *et al.* 2021, Alcock *et al.* 2023, Bonin *et al.* 2023, Gschwind *et al.* 2023, Yin *et al.* 2023). Comparing the outputs of these tools is important in interpreting resistance data from clinical isolates and non-clinical settings (environmental and host-associated microbiomes). However, these outputs use differing nomenclature. This means that a direct comparison between them requires manual labor and considerable domain expertise. For example, *ANT(2ʺ)-Ia*, *ant(2ʺ)-Ia*, *ant(2ʺ)-I*, (AGly)*aadB*, *ANT2-DPRIME*, and 2*ʺ*-aminoglycoside nucleotidyltransferase are different ways to refer to the same gene, but a naïve string comparison would consider these different. ARGs can also have alternative names. For example, *mecA* and PBP2a are different names for the same gene encoding an altered penicillin-binding protein that confers methicillin resistance in *Staphylococcus* spp. (Peacock and Paterson 2015). For large gene families, independent curation by multiple databases can lead to allele numbering discrepancies, despite standardization efforts (e.g. OXA-224 in the CARD is the same sequence as OXA-4 in the DeepARG database).

The same name can even be used for different genes. In 1985, two research groups independently reported and named two different genes *ermA* (Murphy *et al.* 1985, Roberts *et al.* 1985). Despite the fact that both confer resistance to macrolides, these genes have very distinct sequences (only 22% amino acid identity) and are found in different bacterial species. The naming conflict was soon recognized, with sources referring to one of the sequences as *ermR* to disambiguate them as early as 1988 (Kamimiya and Weisblum 1988, Miller 1991). However, the discrepancy persists and currently, the DeepARG database (and others) use the name *ermA* for the ARG that the CARD calls *ermR*.

To facilitate the interpretation of resistance profiles, ARG databases group ARGs into categories. Certain databases categorize ARGs solely based on the antibiotic classes of the drugs they confer resistance to, while others also consider mode of action (e.g. efflux pumps, target modification, and antibiotic inactivation). Furthermore, ARG databases use different nomenclature (e.g. beta-lactam, beta-lactamase, or beta-lactamases) and even implement drug categorization differently (e.g. some define individual beta-lactam groups, such as penems, penams, and carbapenems as separate categories; while others define only the broader beta-lactam group). Altogether, these factors lead to discrepancies in how ARGs are categorized, especially for those that confer resistance through multiple mechanisms or to multiple antibiotic classes.

**Figure 1.**
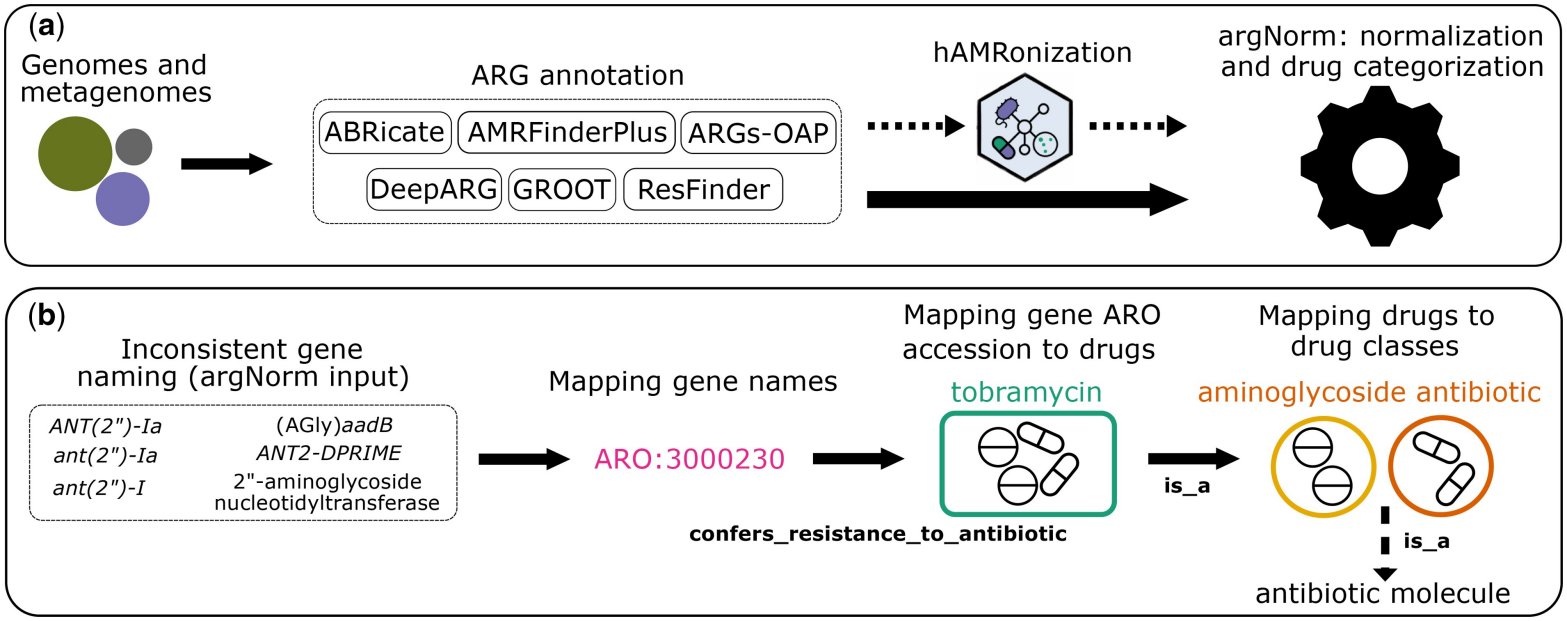
General overview of argNorm. (a) argNorm accepts the outputs of ARG annotation tools (directly or after processing by hAMRonization) and performs ARG normalization and drug categorization. (b) The argNorm workflow includes mapping gene names in the ARG annotation outputs to ARO accessions from prebuilt ARO annotation tables and mapping gene ARO accessions to drugs and drugs classes. To categorize drugs, argNorm traverses the ARO and reports the immediate child of the “antibiotic molecule” node as the drug category (Materials and methods).

To address these inconsistencies in gene naming and categorization, we designed and implemented argNorm, a tool that normalizes the outputs of 6 publicly available and frequently used ARG annotation tools (using eight different databases) to the Antibiotic Resistance Ontology (ARO) developed by the CARD team (Table 1, Fig. 1a) (McArthur *et al.* 2013). The ARO is an ontology [i.e. a controlled vocabulary with formalized relationships forming a graph (Stevens *et al.* 2000)] for the annotation and classification of antibiotic resistance determinants. It describes ARGs and mutations, their products, mechanisms, associated phenotypes, as well as antibiotics and their molecular targets.

**Table 1.** Number of ARGs/AROs and unique antibiotic classes covered by the databases in argNorm version 1.0.

ARG database	ARG annotation tool	No. genes	No. unique AROs	No. manually curated genes	No. antibiotic classes^a^
ARG-ANNOT v5.0	ABRicate v1.0.1	2224	2063	13	19
DeepARG v2	DeepARG v.1.0.2	12 279	2413	197	32
GROOT v1.1.2	GROOT v1.1.2	8948	4814	2	–^b^
MEGARes v3.0	ABRicate v1.01	7784	4721	518	37
NCBI Reference Gene Database v3.12 and v4.0	ABRicate v1.0.1 AMRFinderPlus v3.10.30 and v4.0.19	8957	5921	415	42
ResFinder v4.0	ABRicate v1.0.1 ResFinder v4.0	3150	2449	53	32
ResFinderFG v2.0	ABRicate v1.0.1	3462	350	8	42
SARG (reads mode) v2.0	ARGs-OAP v2.3	12 746	4553	4	30

a These classes also contain metal, biocide, and virulence/stress response gene classes.

b GROOT does not provide drug categorization.

Once ARG annotation results are mapped to ARO accessions, we can use the information available in the ontology to enrich the results of ARG annotation tools in a standardized manner. In particular, as a command-line tool, argNorm adds information to the outputs of ARG annotation tools using the same ARG categorization so that they are comparable, with a key strength being its ability to provide drug categorization of ARGs. As a Python library, argNorm enables the exploration of ARO information associated with ARGs through the pronto library (Larralde *et al.* 2024).

Additionally, argNorm can be run in combination with the hAMRonization package as they perform complementary tasks (Mendes *et al.* 2024). The hAMRonization pipeline standardizes the annotation output formats (by mapping individual output fields to a unified data specification) and argNorm standardizes the nomenclature in the output (by mapping annotated ARGs to unique ARO identifiers for genes, drugs, and drugs classes).

## 2 Materials and methods

### 2.1 Mapping ARGs to ARO terms

ARGs from supported databases were processed through RGI (Resistance Gene Identifier) (Alcock *et al.* 2023), in order to map them to ARO terms. We stored these mappings in a tabular form, which argNorm uses as a reference to map input genes to the ARO.

Specifically, ARG coding sequences were first translated to amino acids, except for sequences from MEGARes. Within MEGARes, the coding sequences are surrounded by non-coding genomic context and, thus, cannot be translated directly. RGI v6.0.3 with the CARD v4.0.0 database was used with the following parameters: “--input_type: protein” (except for MEGARes, where “--input_type: contig” was used), “--alignment_tool: BLAST,” and “--include_loose” (to include loose hits in addition to strict and perfect hits) (Alcock *et al.* 2023). The drug class of each gene is compared to the drug class of its assigned ARO, and in cases where they do not match (even allowing for variations), the gene is flagged for manual curation. Genes that are not automatically mapped by RGI are also manually curated. ARGs that are not present in ARO, were manually mapped to their gene family’s ARO term.

### 2.2 Drug categorization

To obtain information on the drugs to which ARGs confer resistance, argNorm uses the ARO v4.0.0. In particular, argNorm follows the “confers_resistance_to_drug_class” and “confers_resistance_to_antibiotic” relationships in the ARO from the gene of interest, and all its superclasses (as defined by the “is_a” relationship), and all genes related to these superclasses by the “participates_in,” “part_of,” and “regulates” ARO relationships to map genes to drugs. Drugs are classified into drug classes by (i) following the “is_a” relationship to find immediate children of the “antibiotic molecule” node and (ii) additionally following the “has_part” relationship (which is used to link drugs that are mixtures to their components). Drug classes are defined as the immediate children of the “antibiotic molecule” node (i.e. nodes with an “is_a” relationship to “antibiotic molecule”), except for the “antibiotic mixture” node as argNorm returns the constituent drug classes associated with the mixture. A drug may be assigned to more than one drug class (although in the current version of ARO this only happens for the mixture trimethoprim-sulfamethoxazole, which is both a sulfonamide and a diaminopyrimidine antibiotic).

### 2.3 Implementation of argNorm

argNorm is written in the Python language using the pandas (McKinney 2010), BioPython (Cock *et al.* 2009), and the pronto libraries (Larralde *et al.* 2024). argNorm accepts output files directly from supported ARG annotation tools or outputs processed by hAMRonization. argNorm only supports outputs of hAMRonization that were obtained from supported ARG annotation tools. Currently supported tools and versions are listed in Table 1.

argNorm keeps ARO mappings that were automatically derived from RGI separation from manually curated mappings. This reduces the effort required to update the mappings as automated mappings can be easily replaced without overwriting previous manual curation data.

## 3 Results and discussion

We developed argNorm—a command-line tool and Python library—with the goal of normalizing the outputs of ARG annotation tools by mapping ARGs to the ARO. argNorm currently supports six tools and eight databases (Table 1, Fig. 1a). argNorm also supports outputs from the hARMonization package.

argNorm is based on prebuilt mappings from ARGs to ARO terms. These were obtained by running the RGI tool to annotate databases and manually curating any missing hits (Materials and methods). RGI successfully maps the majority of ARGs, with <1% requiring manual curation. However, it is important to note that some databases include genes that are not ARGs (e.g. MEGARes includes metal and biocide resistance genes, and the NCBI Reference Gene Database includes stress response and virulence genes). These non-ARG entries are not part of the ARO and therefore cannot be mapped by argNorm.

argNorm’s output includes additional information on the specific drugs that each ARG confers resistance to as well as their corresponding drug classes, which are derived from the ARO’s hierarchical structure (Fig. 1b). In particular, argNorm reports the immediate child of the “antibiotic molecule” term as the class of each drug. For example, the ANT(2*ʺ*)-Ia gene is associated with resistance to kanamycin A, sisomicin, dibekacin, tobramycin, and gentamicin, all of which are descendants of the “aminoglycoside antibiotic” term in the ARO (Fig. 1b). This approach, although less specific than using all intermediate terms (the ARO has 94 intermediate terms between “antibiotic molecule” and specific drug terms) results in a larger set of classes compared to other databases. For example, while ARG-ANNOT, DeepARG, and MEGARes databases have 19, 32, and 37 drug classes, the ARO provides 62 immediate children of the “antibiotic molecule” node (Table 1).

Mapping between databases that were built using different principles cannot be done exactly. We used RGI to map ARGs to the ARO, which reports results with three levels of stringency: Perfect, Strict, and Loose (Alcock *et al.* 2023). Only 50.8% of the mappings were Perfect, meaning that the exact same amino acid sequence was found in both the original database and CARD—which underlies the ARO. To maximize database coverage, we incorporated all hits (including Loose ones, which make up 21.2% of mappings). Since almost all inputs are assumed to be ARGs, we considered the risk of a complete misidentification to be low and manually assigned correct ARO terms when we identified those cases. Nonetheless, in many cases, the assignment of an ARO term to a sequence is not precise. For example, the *YbxI*|KJ691939.1 gene in the ResFinderFG database was mapped to the ARO term for the *BSU-1* gene. While this is a Loose hit (amino acid identity is 43%), both genes code for class D beta-lactamases.

Despite these challenges, there is a practical need to compare annotations across tools, particularly in large-scale genomic and metagenomic studies. By utilizing the ARO and introducing a consistent categorization of ARGs, argNorm enables the comparison and analysis of results across different annotation tools and databases, contributing to more consistent and comparable research outcomes.

The current implementation of argNorm provides a foundation for standardizing ARG annotation outputs. However, with increased efforts in AMR surveillance, expanding database compatibility beyond the currently supported tools is a critical area for the future development of the tool. Moreover, systematic evaluations of mapping accuracy across different ARGs and mechanisms would help identify areas of improvement and inform future strategies for the normalization of ARG annotation results.

## Data Availability

argNorm is on GitHub at: https://github.com/BigDataBiology/argNorm and on Zenodo at: https://doi.org/10.5281/zenodo.13376001. This manuscript describes argNorm version 1.0 Results shown in this manuscript can also be found on GitHub at: https://github.com/BigDataBiology/argNorm_benchmark. argNorm is also available as a Bioconda package (Grüning *et al.* 2018).
